# Zethembe: a co-developed couples intervention for young heterosexual couples in informal settlements in South Africa

**DOI:** 10.1371/journal.pgph.0004332

**Published:** 2025-04-08

**Authors:** Andrew Gibbs, Zama Khoza, Sivuyile Khaula, Smanga Mkhwanazi, Jenevieve Mannell, Laura Washington

**Affiliations:** 1 Department of Psychology, University of Exeter, Exeter, United Kingdom; 2 Gender and Health Research Unit, South African Medical Research Council, Durban, South Africa; 3 Institute of Global Health, University College London, London, United Kingdom; 4 Centre for Rural Health, University of KwaZulu-Natal, Durban, South Africa; 5 Project Empower, Durban, South Africa; George Mason University College of Public Health, UNITED STATES OF AMERICA

## Abstract

Effective interventions to address violence against women and girls in urban informal settlements are limited. To address this gap, we undertook an intervention co-development process, bringing together four young women (aged 18–25 years) living in an urban informal settlement, academics and NGO partners. Following the 6 Steps in Quality Intervention Development (6SQuID) approach we collaboratively worked through the steps using participatory methods, supporting the young women to think critically and interrogate their lived reality, identify the causes of violence in their lives, and where they felt change was possible. We co-created Zethembe Couples Care, and ‘pre-tested’ this with 17 participants (some were couples). Finally, the academics and practitioners revised the intervention and theory of change. The co-development process led to a series of learnings: the process of building trust and supporting young women to reflect and understand their lived realities took a long time (12 of 15 months), limiting intervention development time. The process also enabled young women to push back against received academic/practitioner wisdom, leading to a couples intervention focused on addressing communication and problem solving, where they felt change was possible, but potentially they could not adequately consider addressing structural drivers of violence. The Zethembe Couples Care intervention now requires piloting at a larger scale to develop it further and formally evaluate it.

## Introduction

Violence against women and girls (VAWG) is common globally [1]. Urban informal settlements – areas where housing has been constructed illegally, with little or no access to basic services – typically have higher rates of VAWG than other communities [2,3], partly driven by poverty and social disorganization. In South Africa, nationally representative data estimated past year intimate partner violence (IPV) prevalence of 15%–17% among women aged 18–44 years [4], and in KwaZulu-Natal Province, where this study is set, past year IPV was 10% [4]. However, in one study in informal settlements in Durban, South Africa, 65% of women reported experiencing IPV in the past 12 months [5].

While there are well evaluated interventions that prevent IPV, including working with women who experience violence, as well as male perpetrators [6,7], there remains little evidence of what works to prevent IPV among younger women living in urban informal settlements [6,8]. Current research suggests a range of reasons for the lack of effective interventions working with young women, which have typically focused on addressing gender norms and strengthening livelihoods [6]. One set of research suggests that young women lack the ‘space’ to create change, given the constraints of poverty and male control they face, while other research suggests the high levels of poor mental health they experience needs addressing first, before wider changes in women’s lives can occur [6,9].

Another potential reason for the lack of effective IPV interventions for young women living in urban informal settlements is that interventions have not been co-developed between young people, practitioners and academics, leading to interventions that do not resonate with young women’s lives [10,11]. This critique parallels criticism of how ‘top-down’ HIV-prevention interventions failed to resonate with the lived realities of intervention beneficiaries, leading to ineffective interventions [12]. Co-development of interventions extends this argument further suggesting that interventions designed by experts focus on ‘risk factors’ for poor health [11], rather than identifying and building on where young women can and do effect actions in their daily lives and strengthening the strategies they already use to create safety [11]. This leads to a mismatch between what intervention participants are offered and where they can enact change in their lives. Co-development, also seeks to create a space where different forms of knowledge, ‘local’, NGO-practitioner and academic, are equally valued [13], creating more effective interventions.

Co-development is not uncontested. The inclusion and roles of ‘potential-beneficiaries’ varies significantly [14]. Moreover, ‘participation’ in these processes maybe partial, with people choosing to remain silent in processes [14]. Additionally, co-development processes are often challenging because of their high cost and long processes [15,16]. Moreover, there remains real concern that power is never equitably held between groups [17].

In this paper we describe the co-development of an intervention to address IPV, working with young women living in urban informal settlements in Durban, South Africa over 15-months (from 2020 into 2021). This was a joint project between Project Empower, a non-governmental organization (NGO) with experience of working with young people these settings, and academics at the South African Medical Research Council and University College London. We describe three steps in the process: i) the recruitment of young women to be involved in the co-development process, ii) the co-development process, and iii) the final intervention and theory of change that emerged.

## Methods and materials

### Site

Urban informal settlements continue to grow in South Africa, with an estimated 520 informal settlements in Durban, translating to approximately 25% of all households [18]. Urban informal settlements are typically a mixture of both ‘formal’ housing (i.e., pre-planned houses built out of bricks and formally provided with basic services) as well as shacks made from wood and metal. Notably informal settlements have little government provision of water and electricity, although the South African government often now provides basic services in the form of temporary toilet and shower blocks.

There is clear evidence that urban informal settlements are spaces where HIV, poor mental health and community violence are common [5]. In South Africa, HIV-prevalence is highest among people living in urban informal settlements [19]. Prior research has also found that almost half of young (18–30 years old) people in informal settlements in Durban may have depression [5].

Young people, however, often move to informal settlements from rural communities to seek new opportunities and escape parental/adult control [20]. This internal migration is often circular, with young people moving between rural homes and urban areas as they search for work for shorter or longer periods returning ‘home’ in between. Young people continue to have high rates of unemployment. In 2020 (prior to the COVID-19 pandemic), it was estimated that among those aged 15–34 years old, 41% were not in employment, education or training [21].

The specific informal settlement community we worked in, in Durban, comprised a few abandoned formal houses, surrounded by shacks, close to the coast and was walking distance to light industry/factories, which offered limited employment opportunities. There was an old, concrete play area, and shared public toilets and showers, though all in a poor state. Water was from standpipes centrally located in the community. There was no easy access to any sports fields or recreation spaces. The majority of people who lived in the community had migrated from rural areas in KwaZulu-Natal, and many lived with siblings or extended family members. A few people lived with their partner. Many young women had their own children as well.

### Project methodology

The project was structured into three phases: recruitment of young women to work with the team, the co-development phase, and a final phase of intervention consolidation.

#### Recruitment of young women.

To identify co-developers we recruited young women from one urban informal settlement, who fitted the inclusion criteria: not currently working or in school and aged between 18 and 25 years old. Once selected they would be hired by Project Empower as Youth Peer Research Associates (YPRAs) and paid a salary for their involvement during the project period. The recruitment process was structured to identify participants who would be committed to the work and recognize its importance beyond their own self-interest. Recruitment comprised workshops and informal discussions often via WhatsApp, over several weeks. During recruitment, there was little follow-up if participants dropped out, as this was seen as an indication that they could not, or did not want to, be involved. Recruitment of young women began on 07/September/2020 and the process ended on 02/October/2020.

#### Co-development process.

The co-development approach was guided by the 6 Steps in Quality Intervention Development (6SQuID) process [22] to develop complex interventions (Table 1). In this project we stopped at step 5 given funding and time constraints. Over 15 months we – YPRAs, Project Empower and academics – sought to complete 6SQuID using multiple methodologies (Table 1). Methodologies were iterative and developed over the project based on previous steps and how the YPRAs responded to activities.

**Table 1:** Methodologies used to complete the 6SQuID Steps in the Zethembe project.

6SquID step	Methodologies used	
1) Identifying the issue and its drivers	Modified photo-elicitation process – with YPRAs taking photographs, sharing them individually with Project Empower via WhatsApp and then followed up with an individual interview to discuss the photos in detail Modified story completion and River of Life mapping process, as well other activities Workshop focused on conflict in relationships using role play Thematic analysis of data generated in the project, using Braun and Clarke [23], which was then fed back to facilitators	Creation of a theory of change – via a problem tree and solution tree – to explain relationships between drivers and issue
2) Identifying what is changeable within this	Workshops discussing theory of change to identify where change is possible	
3) Identifying the mechanism of change	Workshops and discussion groups with YPRAs to identify delivery mechanisms Review of evidence of potentially relevant interventions	
4) Deciding the delivery mechanism		
5) Testing and modification of the intervention	Theatre testing with YPRAs of various intervention sessions, drawn from existing IPV prevention manuals, linked to the theory of change Pre-test of the intervention manual with young people (not YPRAs)	

#### Intervention consolidation.

After completion of the co-development process, the practitioners and academics drafted a final theory of change (ToC) to fully describe the intervention. The ToC drew on a realist evaluation framework [24] describing the mechanisms of impact, explicitly connecting the intervention context, mechanisms of change, and outcome to fully describe how we envisage the intervention impacting on young people’s lives. This allows recognition of how interventions may shape change in people’s lives and have been used to describe violence prevention interventions [25].

Throughout the three steps the team – Project Empower and academics – had weekly phone calls to discuss the project, as well as face-to-face meetings. Two Project Empower facilitators worked closely with the YPRAs. The first facilitator was Black, male and a few years older than the YPRAs, and had extensive experience of facilitation and working in informal settlements. The second facilitator was Black, female and only slightly older than the YPRAs. She had facilitated group activities previously, but not directly with young women from informal settlements. During meetings and co-development processes, we took notes, discussed activities and learnings and reflected on the materials produced (e.g., flipcharts, maps) to enable reflection on the overall project.

### Ethics

The study received ethical approval from the South African Medical Research Council’s ethics committee (EC023-7/2020) as well as UCL’s ethics committee (9663/004). Participants were all over 18 and provided written informed consent prior to participation. Participants had access to a trained psychologist.

## Results

### Recruitment of YPRAs

YPRA recruitment began with a two-day workshop in September 2020, with women accessed via Project Empower’s previous community contacts – beginning 7 September 2020 and ending on 2 October 2020. In total 35 participants arrived at the first workshop, however some were school learners (because there was still rotational learning because of COVID-19 rules) and were asked not to attend the following day. During the workshop, we encouraged women to talk about general issues around their lives in the community.

After the two day workshop we established a WhatsApp group for two weeks. WhatsApp was chosen because it uses little data, is secure and widely used by young people. Using a group chat, Project Empower asked about broad challenges young women faced in the community. At this stage no cellular data were provided. The WhatsApp group was used to gauge people’s ongoing interest in the broad issue as well as their ability to communicate via WhatsApp as the project would use this a lot as a means of communication. While we recognized that this may exclude people without cellphones, or where cellphones were source of conflict and control [26], given we would likely communicate at times via WhatsApp given COVID-19, we needed people to be accessible. By the end of this process six women remained in regular contact. The six were invited to a ‘Beach Day’ a more informal experience, to build the relationship between the young women and the facilitator.

Practitioners and academics finally selected four women who were appointed as YPRAs. The YPRAs were aged between 20 and 24 years old. One had not completed school, two had completed secondary education (matric) and the third had studied for a short while at tertiary level. They were selected to ensure a mix of backgrounds, two had migrated to the informal settlement we were working in, in Durban, after being born in rural KwaZulu-Natal, and rural Eastern Cape. The other two had been born in Durban, but not specifically in the informal settlement we were working in. They also had different personalities, two were more outgoing and confident, and the other two were quieter. YPRAs were not specifically selected for having experienced violence in their intimate relationships, although some had.

YPRAs then went through a two-week orientation programme. The first week focused on providing basic project information, an overview of the methodology, as well as how this work fitted into the broader field of VAWG. The second week sought to build trust and group cohesion, as well as support the women to think more about issues around HIV, VAWG and the challenges young women faced. This drew on ideas from Freire and Boal [27,28], as well as manualized interventions like Stepping Stones [29].

### 6SQuID

#### Step 1 – Identifying the issue and its drivers.

Over twelve months we (YPRAs, Project Empower, academics) worked to co-construct an understanding of the issue (IPV) and its underlying drivers, through developing a ToC. Project Empower supported the YPRAs to think critically and interrogate their lived reality and the challenges they faced, and this also generated ‘new’ data for the academics and practitioners to better understand young women’s lives.

The first activity YPRAs engaged in was an artefact-creation process over the Christmas period (December 2020 to January 2021), which used a modified photo-elicitation approach. In this process, YPRAs were asked to send a Project Empower facilitator photos, messages or voice notes when something interesting occurred – it was up to them to define ‘something interesting’. After the holiday, Project Empower conducted interviews with the YPRAs about their 10 favourite photographs, messages or voice notes. These interviews enabled YPRAs to reflect, as well as provide additional meaning for the practitioners and academics.

YPRAs then completed a modified story completion activity [30], merged with the River of Life exercise from Stepping Stones [29]. In our process YPRAs would tell the life story of a fictional young person like themselves through the metaphor of a river. We (academics and practitioners) anticipated YPRAs may find this easier to externalize issues and talk about challenging experiences, as the group was still building trust. However, creating fictional characters proved challenging. YPRAs struggled to conceptualise alternative lives, quickly slipping back into reflecting on their own lives. YPRAs were also hesitant to share too much of their own River of Live, because of the sensitive information within them, and this group activity quickly became an individual activity with each YPRA working and sharing with a Project Empower facilitator.

The YPRAs also undertook other activities to reflect on their lives and provide data for the academics. These included creating timelines of their weekly activities and mapping important (intimate and non-intimate) relationships in their lives. During each activity there was a chance to discuss what was found as a group and Project Empower supported the YPRAs to reflect on their lives, and the facilitators encouraged the gendered nature of women’s lives to be discussed.

Given the project’s central focus on IPV we (academics and practitioners) felt YPRAs needed to directly reflect on conflict in intimate relationships. YPRAs were asked to create four scenarios, two showing conflictual relationships and two showing good relationships. YPRAs decided for themselves what a conflictual or good relationship was. These scenarios were then acted out and filmed and then re-watched and discussed, reflecting a ‘theatre of the oppressed’ methodology [27], with the facilitator emphasizing the need to consider the role of ‘gender’. This allowed focused discussion on strategies young women use to navigate conflict. However, the YPRAs struggled to imagine ‘good’ relationships among peers, instead role-playing idealized older married couples, not living in their community, as their only reference to ‘good’ relationships.

During these reflection/data collection processes, the YPRAs were supported to construct a ToC to map how the issues they had identified were linked to IPV. To provide a visual representation this was done using a ‘problem tree’ approach [31,32]. In a problem tree analysis, the trunk is the problem (IPV), the roots the underlying causes, and the leaves/branches the outcomes. The problem tree for the YPRAs (see S1 Fig) was developed over several sessions. The first problem tree was (in our – academic and practitioner – view) too broad and non-specific (e.g., it had the ‘cellphone’ as a problem, rather than more clearly spelling out why ‘cellphone’ could be linked to IPV). As such at the request of the practitioners and academics this was re-done, focusing on how underlying factors were connected to IPV.

Finally, the data the YPRAs had produced were ‘formally’ analysed by the academic researchers on the team. The researchers used thematic analysis [23] to rapidly synthesise the YPRAs data focused on ‘risk’ and ‘safety strategies’ and then created a series of mind-maps reflecting these. This was then presented back to the YPRAs to review. YPRAs typically agreed with the analysis (though not always) and made some minor modifications to their ToC following this. During feedback to the Project Empower facilitator, the YPRAs mentioned how this process enabled them to see how the activities they had done were connected into the wider project, giving them a sense of forward progress and connection.

#### Step 2 – Modifiable risk factors.

To identify modifiable risk factors we conducted a series of workshops. First, drawing on the YPRA problem tree (S1 Fig), we rotated it to a ‘solution tree’ (S2 Fig) and discussed with the YPRAs what could be done to address the underlying root causes in the problem tree. YPRAs identified a wide of potential solutions, ranging from solutions they had no control over (e.g., increasing access to sports grounds and parks, which would have required government intervention), to those that they felt women like themselves could enact in their own lives (e.g., conversations). Discussion between the YPRAs and Project Empower led to a focus on three specific areas: conflict solving skills, communication, and parenting skills. YPRAs felt these were important for them, and that they could acted on them.

#### Step 3 – Identifying the mechanism of change and Step 4 identifying delivery mechanism.

Step 3 and Step 4 of the 6SQuID process merged. The mechanism of change refers to the active ingredients of the intervention, essentially the theory of behaviour change, while the delivery mechanism refers to how the active ingredients will be delivered (e.g., groups, individuals). It was challenging to disentangle these steps while working with the YPRAs, as the delivery mechanism also effected the mechanism of change.

Through ongoing discussions with the YPRAs it became clear that they felt the identified modifiable risk factors, conflict solving skills, communication and parenting skills, were all issues that they felt were best addressed in collaboration with their male partner. When previously creating interventions for young people from urban informal settlements we, the academics and practitioners, had shied away from couples interventions for this group, given research highlighting young people’s fluid, multiple and complex intimate relationships in these settings [33,34]. Moreover, many IPV prevention interventions focused on couples required couples to be married or living together for at least six months [e.g., 35], which was uncommon among young people living in urban informal settlements. Through discussion and reflection and upon listening to the logic YPRAs put forward for this focus, we agreed to have the intervention focus on couples.

YPRAs also felt an intervention needed to be short, ideally 5 sessions, each lasting 2–3 hours, to ensure attendance. Again, this contrasted sharply with our previous work, where interventions were long – for instance the Stepping Stones and Creating Futures intervention was 63 hours contact time (11 sessions, 3 hours/session), which caused challenges in participant attendance [36]. Again, we (academics and practitioners) reflected on this and agreed that the aim was to create an intervention which supported young people rather than impose our solutions.

Based on the identified modifiable risk factors and the need for a couples focused, short intervention, the academic and practitioner team conducted a rapid review of interventions effective at reducing violence working with couples, or that focused on communication skills, problem solving, or addressed children in a couples’ relationship. To do this we searched for published papers and reviews, discussed with experts and looked for available manuals addressing these topics, and identified five potential sessions from effective interventions. The five sessions included one from Stepping Stones [29] on gendered expectations in relationships. Stepping Stones has shown a wide range of positive benefits around transforming gender norms and relationships [37]. Another session came from Indashikyiwra and focused on healthy relationships and understanding emotions [35]. Indashikyiwra was evaluated in Rwanda with heterosexual couples who lived together, and demonstrated significant reductions in IPV [35]. A third drew from Bandebereho, an intervention to promote male engagement around children [38], which was effective at reducing IPV, and the fourth used problem solving methodologies [39], which have been shown to be effective in addressing low mood [40].

#### Step 5 - Testing and modification of the intervention.

Testing and modification of the intervention was done in two ways, first with the YPRAs and second with other young people.

#### Theatre testing.

Theatre testing of sessions enabled quick feedback from YPRAs. As the YPRAs were all women we drew in some young men from the same community as the YPRAs – who were not the YPRAs’ partners, but of a similar age to YPRAs (22 to 30 years old). After each session we discussed what they had liked and what they had not, and the reasons why. At the end of all sessions the group was split into two (men/women) for a final discussion about the sessions.

Theatre testing identified some challenges with the sessions. The session on raising children was challenging to run. YPRAs expressed they often did not see their children, as they were cared for by others, often grandparents in rural communities and it was agreed the session should be dropped. During the session focused on problem management skills, YPRAs spoke generally about challenges they identified, rather than specific challenges. As sessions progressed people spoke more specifically about their issues, and the problem-solving activity was moved into a ‘homework’ exercise. Finally, based on feedback from YPRAs we introduced a ‘couple care jar’ as they wanted to continue exercises outside of the intervention space. The couple care jar provided simple topics couples could talk about; gestures of appreciation they could do for their partners, activities they could do together and encouraged openness about sex, and were all low or no cost.

The delivery mechanism was also finalized during the theatre testing. It was agreed with the YPRAs that sessions would be delivered by two facilitators (a male and female) working together. There would be a maximum of five sessions, and each session would be 2.5 hours long, ideally spread over three-weeks. Sessions would be delivered in the community with young people (aged 18–25 years).

#### ‘Pre-test’.

To provide further feedback and modification of the intervention we conducted a small ‘pre-test’ of the intervention with young people. YPRAs were asked to recruit heterosexual couples from their community, where the woman was aged 18–25 years, out-of-school and not in formal work. The venue for the intervention was a small informal *shebeen*, which in the evenings sold alcohol, but was ‘closed’ during the late morning and afternoon when the intervention was delivered. Rather than being delivered over a three-week period the intervention was delivered over a hot week in January 2021, because of concerns about a COVID-19 spike emerging and new lock-down rules being imposed. Participants received R200 (~£8.70) for attending each session.

Overall, 17 people came to the first day (9 women and 8 men), with most of them being aged 18–25 (and all over 18). Four were ‘genuine’ couples (i.e., 8 people) and one of these ‘genuine’ couples had a small baby, only a few months old. The others who came appeared to be pretending to be in a relationship, which the facilitators identified through the ‘couples’ not really knowing details about each other’s lives, not having an easy rapport and so forth. On the second day another ‘real’ couple started to attend. Throughout the week there was a gradual reduction in attendance and on the last day 10 people remained (3 couples, plus an additional four). Some young men got called in at short notice for piece work (causal, informal work), and some young women had other commitments.

The pre-test provided several key learnings leading to modification of the ToC and intervention. First, young couples valued the space the intervention created, as they rarely had a chance to discuss their relationship or clarify their expectations. Second, participants valued the ‘cognitive behavioural triangle’ (thoughts, feelings, actions), which was part of one of the sessions, as a tool that helped them to clarify their feelings and behaviours and provided an opportunity to discuss issues and challenges that they faced in their life. However, they struggled to discuss emotions, especially positive emotions and facilitators spent a long time on this and in the revision more time was allocated to a focus on positive emotion. Third, there was a strong expectation of monogamy in relationships, but this was rarely the actual case. This disjuncture between norms of monogamy and the reality of multiple-partnerships meant during intervention activities there were silences and gaps, and we needed to more directly tackle this. Finally, the problem-solving activity ‘homework’ was not undertaken and needed to be reintroduced as a standalone session.

### Intervention consolidation

Based on feedback from the pre-test the academic and practitioner team revised the intervention and revised a ToC for the intervention. The formalization enables future piloting of the intervention (Step 6 of 6SQuID). The revised ToC for Zethembe Couples Care (S3 Fig) emphasises how structural realities impact on young people’s social and intimate relationships, so that intimate relationships become sites of conflict. Conflict was shaped by lack of trust and communication, and limited ability for joint problem solving, as well as male domination of decision making. In response the intervention sought to address three areas:

1) Clarifying relationship expectations – three sessions focusing explicitly on discussing gendered expectations in relationships and working with couples to identify what healthy relationships could look like. This included an emotional, as well as practical, focus on relationships.
2) Strengthening communication skills – learning non-violent and non-aggressive ways of communicating are central to effective VAWG prevention programming [6]. Communication skills included learning communication strategies, and the role of emotions in shaping communication.
3) Conflict resolution skills – focused on problem solving therapies [39]. Based on the pre-test a full session was included enabling couples to practice skills to work together to solve a non-threatening problem in their relationship.

## Discussion

We have described the process of co-developing an intervention with young women living in urban informal settlements. In this project, young women, worked closely with the practitioners and academics to co-develop an intervention merging YPRAs understandings of where and how they saw change as possible, with practitioners and academics scientific and practical knowledge of what may make effective IPV prevention interventions. This culminated in the Zethembe Couples Care intervention. In this discussion we reflect on the benefits and challenges of co-development as a process.

The co-development process enabled YPRAs to challenge practitioner/academic knowledge, most clearly that the intervention target couples. The co-construction of knowledge and the contestation of academic knowledge is central to co-development [13,41] reorientating relationships of power [41]. In this process the academic/practitioner team reflected and modified their stance based on discussions and feedback from the YPRAs, integrating the YPRAs perspectives and understandings.

Despite this fusion of knowledge and co-creation of a couples intervention, during the pre-test a number of challenges emerged. Few ‘proper’ couples attended. Moreover, young people’s complex intimate relationships, with overlapping and multiple partners, meant discussions in sessions were often fraught. Some of these challenges were part of the ‘modification’ and refinement process and expected in any intervention development process. However, it could also be that we relied too heavily on ‘lived experience’ without adequate integration of the practitioner and academic knowledge.

The co-developed intervention addressed factors the YPRAs felt were important and amenable to change, specifically communication and relationship skills focused on the couple dyad. This suggests that co-development can highlight spaces of agency available to people, which are not seen often in typical research processes [11]. In contrast previous interventions for young women in informal settlements have been more focused on addressing women’s structural disempowerment, particularly poverty, but have not been successful at reducing women’s own experiences of IPV [6,8,11]. The different intervention emphasizes could be linked to ‘bounded rationality’ [42] – YPRAs made choices and assessments about the intervention based on the information available to them, and thus focused on small wins in spaces where they had some control. Yet, in privileging this analysis and approach to intervention, we may have downplayed the importance of tackling structural drivers, and forgotten the importance of how multiple perspectives can be used to understand the issue [41] and potential solutions.

Throughout the process of co-development it took a long time to build trust among the YPRAs and between the YRPAs and facilitators, trust is critical for an effective co-development process [43]. This may have been a function of the ongoing COVID-19 lockdowns during the project, the nature of the topic, which led to discussions deemed very sensitive and/or the YPRAs own lack of trust given their experiences in the past. The long length of time for co-development caused problems as it limited the time for intervention development.

A final challenge was that despite this being a co-development process we, the academic/practitioner team, established and controlled the overall process. We set activities and timelines and made final decisions about the Zethembe Couples Care intervention, including the ToC. The challenge of equally sharing power in co-development processes is clearly recognized [17], and is partly driven by project/funding timelines [16], but also reflects the ongoing challenge of co-development.

This study has a series of limitations and strengths. We did not conduct a formal process evaluation of the co-development experience, which would have included interviews with all people involved (YPRAs, academics and practitioners) to understand their experience of this process. Moreover, we worked with a small number of YPRAs reducing the generalizability of their views, which may have limited our understanding of young women’s lives. However, as we worked with a small number of YPRAs over time, this allowed for much greater depth and reflection by the YPRAs, moreover, we – academics and practitioners – could draw on our prior research to provide a wider context and understanding. Despite these limitations the study has strengths, we worked closely with YPRAs over the project period in detailed ways, responding to their issues and understanding of the topic and have sought to emphasise both the positives and negatives of this approach in this analysis.

## Conclusion

The process to co-create Zethembe Couples Care between young women, practitioners and academics while achieving some ideals of co-development, including young people challenging academic knowledge and the intervention seeking to build on spaces where young women felt they had agency, also highlighted the challenges inherent in such processes. These challenges included the continued agenda setting by the academic/practitioner team and the potential over emphasis of ‘local’ knowledge, rather than a fusion of scientific and local knowledge. Future work needs to complete the sixth step of 6SQuID, to ascertain if Zethembe Couples Care demonstrates it is feasible to deliver, acceptable to participants and that there is adequate evidence of effect, before moving on to conduct a formal evaluation of the intervention to see if the intervention can reduce women’s experiences of IPV.

## Supporting information

S1 Fig Co-developed theory of change for Zethembe
(DOCX)

S2 Fig Identification of where change may be possible in young women’s lives
(DOCX)

S3 Fig Theory of Change for Zethembe Couples Care
(DOCX)
