# Supplementary figures and images for: Zethembe: a co-developed couples intervention for young heterosexual couples in informal settlements in South Africa
Source: PLOS Glob Public Health. 2025 Apr 8;5(4):e0004332. doi: 10.1371/journal.pgph.0004332 (PMC11978062; doi:10.1371/journal.pgph.0004332)

**S1 Fig: Co-developed theory of change for Zethembe**

**
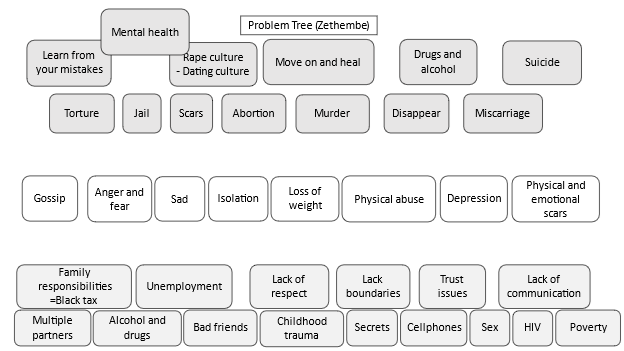
**

Supplement: S1 Fig — Co-developed theory of change for Zethembe (DOCX) [file pgph.0004332.s001.docx]

**S2 Fig: Identification of where change may be possible in young women’s lives**


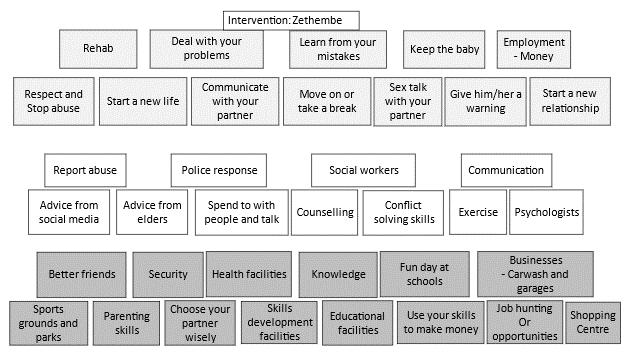

Supplement: S2 Fig — Identification of where change may be possible in young women’s lives (DOCX) [file pgph.0004332.s002.docx]
