# Supplementary material for: Zethembe: a co-developed couples intervention for young heterosexual couples in informal settlements in South Africa
Source: PLOS Glob Public Health. 2025 Apr 8;5(4):e0004332. doi: 10.1371/journal.pgph.0004332 (PMC11978062; doi:10.1371/journal.pgph.0004332)
Supplement: S3 Fig — Theory of Change for Zethembe Couples Care (DOCX) [file pgph.0004332.s003.docx]

**S3 Fig: Theory of Change for Zethembe Couples Care**
